# Ultrasound-guided 5-in-1 trigger point injection for treating tension-type headache: A case report

**DOI:** 10.1097/MD.0000000000029987

**Published:** 2022-08-05

**Authors:** Jun Young Kim, Yoo Jin Choo, Min Cheol Chang

**Affiliations:** Department of Physical Medicine and Rehabilitation, College of Medicine, Yeungnam University, Daegu, Republic of Korea.

**Keywords:** myofascial pain syndrome, pain, injection, headache, ultrasound

## Abstract

**Rationale:**

Tension-type headache (TTH) is the most common type of primary headache, and trigger point injection (TPI) is frequently used for controlling pain originating from TTHs. In the current report, we introduce a TPI technique involving 4 neck muscles (upper trapezius, splenius capitis, semispinalis capitis, and inferior oblique capitis) and a greater occipital nerve (GON) block within the same sonographic view for the treatment of TTHs.

**Patient concerns:**

A 44-year-old woman complained with pressing and tightening, nonpulsating, recurrent headaches, mainly in the bilateral occipital area, lasting for approximately 6 months (numeric rating scale: 5). The patient had no nausea, vomiting, photophobia, or phonophobia.

**Diagnoses:**

The patient was diagnosed as having a TTH.

**Interventions:**

Under ultrasound (US) guidance, a mixed solution of 2 mL of 2% lidocaine and 5 mL of normal saline was injected layer-by-layer into the 4 target muscles of the neck (upper trapezius, splenius capitis, semispinalis capitis, and inferior oblique capitis) and near the right GON within the same sonographic view bilaterally.

**Outcomes:**

Two- and 4-week follow-ups after administration of the injections revealed no headache. Our US-guided 5-in-1 TPI technique is viable for treating patients with TTH.

**Lessons:**

We believe that it can aid in reducing the procedure time and associated pain.

## 1. Introduction

Tension-type headache (TTH) is the most common type of primary headache. Approximately 40% of individuals with headaches have TTH.^[1]^ The most prominent finding in TTH is the presence of mechanical pain hypersensitivity in the muscles of the head and neck.^[1]^ Trigger points of the posterior neck muscles, such as the upper trapezius, splenius capitis, semispinalis capitis, and inferior oblique capitis muscles, are closely associated with the development of TTH.^[1]^ Therefore, trigger point injection (TPI) on the neck muscles is often used for the treatment of TTH and many studies have demonstrated that it has a positive therapeutic effect on TTH.^[1,2]^ In addition, the greater occipital nerve (GON) courses through several layers on the neck muscles, such as the semispinalis capitis and trapezius muscles.^[3]^ Tightened neck muscles in patients with TTH can compress the GON and contribute to headaches in the occipital area.^[3]^ The pain-reducing effect of a GON block in TTH was reported in some previous studies.^[3,4]^

In the posterior neck, several muscles, such as the upper trapezius, splenius capitis, semispinalis capitis, and inferior oblique capitis, are located close together. Therefore, administering an even treatment of all these muscles using a blind technique is not easy. Moreover, TPI and nerve block with a blind technique in the neck area can cause various complications such as spinal cord injury and peripheral nerve or vascular injury. Ultrasound (US)-guided TPI and nerve block have been described as techniques that allow accurate injection of the targeted muscles with enhanced needle visualization and prevention of complications.

Here, we introduce a TPI technique in 4 neck muscles (upper trapezius, splenius capitis, semispinalis capitis, and inferior oblique capitis) and GON block with the same sonographic view for the treatment of TTH. We present the following case in accordance with the CARE reporting checklist.

## 2. Case report

A 44-year-old woman visited the rehabilitation department of our hospital on November 1, 2021, with pressing and tightening, nonpulsating, recurrent headaches, mainly in the bilateral occipital area, lasting for approximately 6 months. The pain was rated as 5 using a numeric rating scale (NRS). When her headache worsened, it usually lasted for 1 to 7 days. The patient had no nausea, vomiting, photophobia, or phonophobia. Physical examination and magnetic resonance imaging of the brain revealed no abnormalities. The patient was diagnosed with TTH and was prescribed oral pain medication (meloxicam 15 mg, acetaminophen/tramadol hydrochloride 650 mg/75 mg, and nortriptyline 10 mg). After treatment with oral pain medication, the initial NRS score of 5 reduced to 4. Because the patient still complained of persistent headache, we performed ultrasound US-guided TPI on the posterior neck muscles.

**Figure 1.**
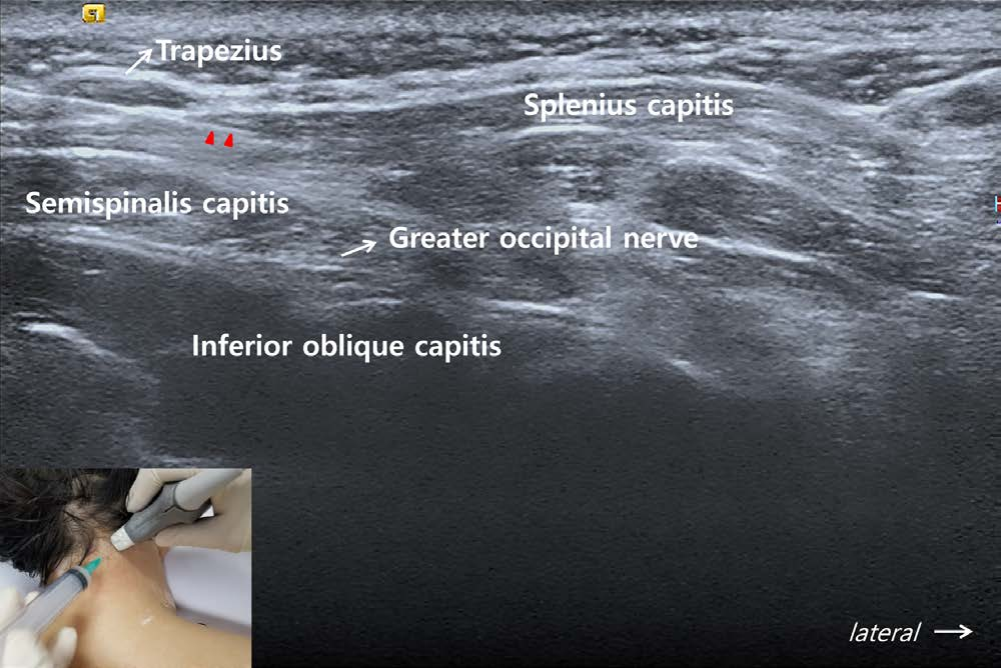
Ultrasound-guided TPI into 4 muscles of the posterior neck area and block of the greater occipital nerve. The US probe was placed on the imaginary line connecting the right spinous process of C2 and the right mastoid process. The right upper trapezius, splenius capitis, semispinalis capitis, and inferior oblique capitis muscles together with the greater occipital nerve are seen. The needle (arrowheads) for TPI is shown inserted into the splenius capitis muscle. TPI = trigger point injection.

Under US guidance (18-MHz linear transducer; S2000; Siemens, Seoul, South Korea), TPI was performed with the patient in the prone position (Fig. 1, see Video, Supplemental Digital Content, http://links.lww.com/MD/G989, which shows US-guided 5-in-1 TPI technique). The US probe was placed on the imaginary line connecting the right spinous process of C2 and the right mastoid process, allowing the identification of the upper trapezius, splenius capitis, semispinalis capitis, inferior oblique capitis, and GON, which was located above the inferior oblique capitis muscle. A mixed solution of 2 mL of 2% lidocaine and 5 mL of normal saline was injected layer-by-layer into the 4 muscles of the right neck and near the right GON within the same sonographic view. We also administered the same injection on the left neck muscles and left GON. Ten minutes after the injection was administered, the pain almost disappeared. Two- and 4-week follow-ups after administration of the injection revealed no headache. The patient reported no adverse or unanticipated events after administration of the injection.

The procedures performed in this study were in accordance with the ethical standards of the institutional and national research committee and the Declaration of Helsinki (as revised in 2013). The study was approved by the local institutional review board of Yeungnam University hospital. Written informed consent was obtained from the patient for publication of this case report and accompanying images.

## 3. Discussion

In this report, we describe the successful treatment of TTH using US-guided TPI on the bilateral upper trapezius, splenius capitis, semispinalis capitis, and inferior oblique capitis muscles, together with bilateral GON block. The procedure was performed on each side as a single injection under US guidance within a single sonographic view.

Active trigger points and palpable nodules in the taut bands of the skeletal muscles in the pericranial or neck muscles cause TTH.^[5]^ Direct injection of anesthetics or dry needling into these trigger points relaxes contraction or spasm and increases blood circulation in taut bands, which contributes to the reduction of pain from TTH.^[5]^ In clinical practice, among the several types of headaches, TPI is most frequently performed in TTH.^[6]^ Several previous studies have demonstrated the positive therapeutic effect of TPI on pericranial muscles.^[1,7,8]^ After TPI, the severity and frequency of headaches significantly reduced. Likewise, several previous studies have also demonstrated the pain-reducing effect of GON block in patients with TTH.^[9,10]^ GON is the branch of the second cervical root and primary sensory nerve of the occipital area of the skull.^[11]^ Blocks with local anesthetics inhibit the transmission of pain signals and reduce ectopic discharge in nociceptive C-fibers.^[11]^

Recently, injections for controlling pain are often administered under US guidance as this allows the procedure to be performed safely without complications such as hematoma and nerve or vascular injury. In addition, US guidance allows accurate injection into the targeted structures. Regarding previous studies on US-guided TPI, most administered TPI to only 1 muscle per injection.^[12–14]^ However, because trigger points develop in several muscles in the pericranial or neck areas, the simultaneous treatment of these muscles enhances the therapeutic effects of TPI. Moreover, when administering TPI to 1 muscle with a single injection, the position of the US probe must be moved every time the muscle is treated. This increases the procedural time and the associated risk of infection.^[15]^ We believe that TPI in multiple muscles with a single injection within a single US plane can overcome the disadvantages of pre-existing US-guided TPI methods.

## 4. Conclusion

We administered TPI to 4 neck muscles (upper trapezius, splenius capitis, semispinalis capitis, and inferior oblique capitis) and GON block with the same sonographic view. Our US-guided 5-in-1 TPI technique reduced the procedure time and pain during the procedure. We believe that our technique will be useful in treating patients with TTH. However, our report is limited by the fact that it was a single case study. In the future, well-designed clinical studies involving larger numbers of patients with TTH are required to demonstrate the usefulness of our TPI technique.

## Author contributions

All authors approved the final manuscript as submitted and agree to be accountable for all aspects of the work.

Conceptualization: Jun Young Kim; Yoo Jin Choo; Min Cheol Chang

Data curation: Jun Young Kim; Yoo Jin Choo; Min Cheol Chang

Investigation: Jun Young Kim; Yoo Jin Choo; Min Cheol Chang

Supervision: Min Cheol Chang

Writing—original draft: Jun Young Kim; Yoo Jin Choo; Min Cheol Chang

Writing—review and editing: Jun Young Kim; Yoo Jin Choo; Min Cheol Chang

## Supplementary Material


